# The comparison of the degree of apoptosis in ovaries and fallopian tubes between two different surgical interventions for tubal ligation: A rat model

**DOI:** 10.4274/jtgga.2016.0192

**Published:** 2018-03-01

**Authors:** Sezcan Mümüşoğlu, Servet Hacıvelioğlu, Lale Karakoç Sökmensüer, Rengin Karataylı, Ayşegül Süzer, Figen Kaymaz

**Affiliations:** 1 Department of Obstetrics and Gynecology, Hacettepe University School of Medicine, Ankara, Turkey; 2 Department of Obstetrics and Gynecology, Çanakkale Onsekiz Mart University School of Medicine, Çanakkale, Turkey; 3 Department of Histology and Embryology, Hacettepe University School of Medicine, Ankara, Turkey; 4 Department of Obstetrics and Gynecology, Necmettin Erbakan University School of Medicine, Konya, Turkey

**Keywords:** Tubal ligation, bipolar electrocauterization, Pomeroy, ovarian reserve, fallopian tube

## Abstract

**Objective:**

To compare the degree of apoptosis in ovaries and tubal epithelium observed secondary to tubal ligation either by Pomeroy’s method or bipolar electrocauterization in a rat model.

**Material and Methods:**

A total of 24 female Sprague-Dawley rats were randomly assigned into 3 study groups: control (n=8), Pomeroy (n=8), and the electrocauterization group (n=8). Apoptotic cells were detected on the primary, secondary, tertiary follicles of the ovaries, and on the tubal epithelium using terminal deoxynucleotidyl transferase-mediated deoxyuridine triphosphate nick end-labeling. The apoptotic index was calculated for each group by the percentage of the stained cells.

**Results:**

The apoptotic index of tubal epithelium was significantly higher in the bipolar electrocauterization group compared with the control and Pomeroy groups (3.1±0.8 vs. 1.4±1.0, p=0.018 and 2.0±1.2, p=0.03, respectively) whereas there was no significant difference between Pomeroy’s method and the control group. The apoptotic index of primary follicles was higher in the bipolar electrocauterization group compared with the control and Pomeroy’s method groups (3.4±0.5 vs. 1.2±0.4, p<0.001 and 1.8±0.8, p=0.005, respectively), but there was no significant difference between Pomeroy’s method and the control group. The apoptotic index of secondary and tertiary follicles was similar for each group.

**Conclusion:**

Pomeroy’s technique, as a permanent contraception method, is associated with lower apoptotic index on ovary and fallopian tube when compared with bipolar electrocauterization.

## Introduction

Tubal sterilization is a widely used contraceptive procedure with a high efficacy. More than 190 million women around the globe have elected to undergo surgical sterilization as a safe and reliable method of permanent contraception, and it is the second most preferred method in the United States (1).

Despite the advantages of permanent contraception, some concerns have been raised about its complications. Tubal sterilization has been accused of several adverse effects such as irregular menstrual cycles, dysmenorrhea, and climacteric symptoms (2).

The possible explanation of these complications was initially hypothesized as the disturbed vascularization of the ovaries, which subsequently interfered with the ovarian cycle and metabolism (3). There are several studies in the literature reporting the effect of tubal sterilization on ovarian function, follicular development, hormonal levels, and ovarian blood supply (4,5). However, there is still no consensus as to whether changes in ovarian blood flow due to tubal ligation cause damage in ovaries and fallopian tubes. There is a lack of evidence at this time to understand whether women undergoing sterilization will experience an earlier onset of menopause. In addition to these uncertainties about the effect of tubal ligation on ovarian and tubal blood flow, it is also unknown whether the type of surgical technique per se plays role in disturbed ovarian vascularization and ovarian reserve. 

The aims of the current study were two-fold; i) to compare the apoptotic index of ovarian follicles between Pomeroy's technique and bipolar electrocauterization, ii) to compare the apoptotic index of the tubal epithelium between these techniques.

## Material and Methods

### Ethical statement

The Animal Research Ethics Committee of Hacettepe University approved the study protocol by (Approval no: 2012/16). The “Principles of laboratory animal care” (NIH publication no. 86-23, revised 1985) and specific national laws were followed throughout the study.

### Study design and surgical procedure

A total of 24 female 16-week-old adult, non-pregnant Sprague-Dawley rats, weighing between 250 and 300 g were included in this experimental study. All animals were provided by Hacettepe University, Animal Research Center, and they were randomly divided into 3 experimental groups; control (n=8), Pomeroy’s method (n=8), and the bipolar electrocauterization group (n=8). All rats were kept in a positive pressure room, which was equipped with high-efficiency particulate air filter, in filter-topped cages. Unlimited access to food and water was provided during the 12-hour light-dark conditions. 

Anesthesia was obtained via intraperitoneal administration of ketamine hydrochloride 75 mg/kg (Ketalar; Eczacıbaşı, İstanbul, Turkey) and xylazine hydrochloride 10 mg/kg (Rompun; Bayer Türk İlaç Ltd., İstanbul, Turkey). The abdominal wall was cleaned with povidone-iodine (Baticon; Drogsan, Turkey) after being shaved. Surgery was performed on a 37 °C heated warming plate after covering the surgical site with a sterile towel, and laparotomies were performed through a midline incision.

No intervention was made on the control group. In the Pomeroy’s method group, rats received bilateral tubal ligation with Pomeroy’s technique; after elevating the tube, the loop was ligated with a 2/0 Vicryl suture (Johnson & Johnson, USA) 2 cm away from the ovary (6). In the bipolar electrocauterization group, the tube was cauterized by bipolar electrocoagulation 2 cm away from the ovary and interrupted via surgical scissors. After the procedures, the abdominal wall was closed layer-to-layer with 2/0 Vicryl suture (Johnson & Johnson, USA) in each group.

During the recovery period after the first surgery, all animals were kept under the above-mentioned conditions in the same animal research laboratory at Hacettepe University. Fifteen days after the first surgery, a second laparotomy was performed to each rat under intraperitoneal anesthesia and bilateral oophorectomy and salpingectomy was performed within a mean surgical time of 10 minutes. After completing the second surgical procedure, all rats were sacrificed via cervical dislocation. In each group, apoptotic cells on the primary, secondary, and tertiary follicles of ovaries and on the tubal epithelium were stained using terminal deoxynucleotidyl transferase (TdT)-mediated deoxyuridine triphosphate nick end-labeling (TUNEL).

### Histology and TUNEL assay

Standard paraffin wax embedding was used. Specimens were fixed in 10% buffered formaldehyde, dehydrated in a graded ethanol series, cleared in xylene, and embedded in paraffin wax. The paraffin blocks were sectioned using a sledge microtome (Leica Microsystems, Germany) with 5-µm thickness.

Apoptosis of follicular cells and tubal epithelial cells was assessed using enzymatic labeling of DNA-strand breaks with a TdT-mediated TUNEL assay with a Cell Death Detection Kit (Roche, cat no: 11 684 817910) in accordance with the manufacturer’s instructions. In this technique, the TdT binds to the 3’-OH end and synthesizes a polynucleotide at the nick end. The biotinylated nucleotides then interact with avidin-peroxidase, which can be detected histochemically.

All slides were examined under a Leica DM6000B microscope and photographed using a Leica DC490 digital camera.

A positive control and a negative control for the detection of DNA fragmentation were also performed as previously described (7).

### Apoptotic index

All preparates were evaluated by a blinded, experienced histologist (LKS). Epithelium cells in the fallopian tube sections and only the granulosa cells in the ovarian sections were counted. As a counting procedure: i) five random areas in the fallopian tube sections were photographed under x40 magnification and 100 epithelium cells were counted in total; ii) in the ovarian tissue sections, 100 granulosa cells were counted for each type of follicle (primary, secondary, and tertiary). The apoptotic index was calculated by the percentage of the positively-stained cells under microscopic examination of the ovarian tissue and fallopian tube following staining with TdT. According to the apoptotic index, the absence of stained cells corresponded to 0 points; stained cells of 1-25% corresponded to 1 point; 26-50% to 2 points; 51-75% to 3 points, and 76-100% to 4 points. The apoptotic index was compared between the three groups.

### Statistical analysis

All statistical analysis were performed using SPSS, version 16.0 (SPSS, Chicago, IL, USA). The comparison of scores between groups was performed using the Kruskal-Wallis test. Post-hoc analysis was performed using Tukey’s procedure to calculate the difference between groups. Values are given as means, standard deviation, and p<0.05 was accepted as statistically significant.

## Results

**Figure 1:**
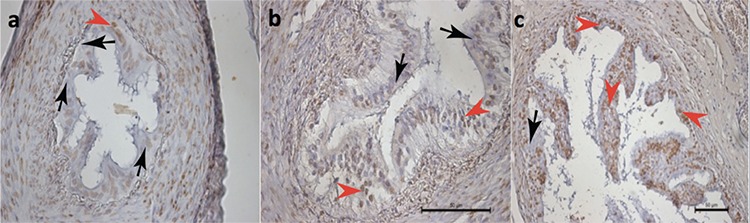
“>” denotes apoptotic epithelial cells in histologic section of fallopian tubes of the rats stained with ApopTag (brown stain), “>” denotes non-stained normal epithelial cells; a) control group, b) Pomeroy group, c) bipolar electrocauterization group (TUNEL assay x400)

There was a statistically significant difference between groups regarding the apoptotic index in the tubal epithelium (Figure 1). In binary comparisons, the bipolar electrocauterization group had a significantly higher apoptotic index compared with the control and Pomeroy’s method groups (3.1±0.8 vs. 1.4±1.0, p=0.018 and 2.0±1.2, p=0.03, respectively), whereas there was no significant difference between the Pomeroy’s method and control group (2.0±1.2 vs. 1.4±1.0, p>0.05) (Table 1).

**Figure 2:**
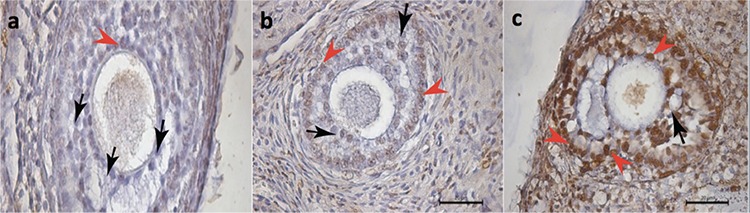
“>” denotes apoptotic granulosa cells of primary follicles in histologic sections of the rat ovaries stained with ApopTag (brown stain), “>” denotes non-stained granulosa cells of the primary follicles; a) control group, b) Pomeroy group, c) bipolar electrocauterization group (TUNEL assay x630)

Similarly, the apoptotic index detected on the primary follicles of the ovaries indicated a significant difference between groups (Figure 2). In binary comparisons, the bipolar electrocauterization group had significantly increased apoptotic index compared with the control and Pomeroy’s method groups (3.4±0.5 vs. 1.2±0.4, p<0.001 and 1.8±0.8, p=0.005, respectively), whereas there was no significant difference between the Pomeroy’s method and control group regarding apoptotic index (1.8±0.8 vs. 1.2±0.4, p>0.05) (Table 1).

**Figure 3:**
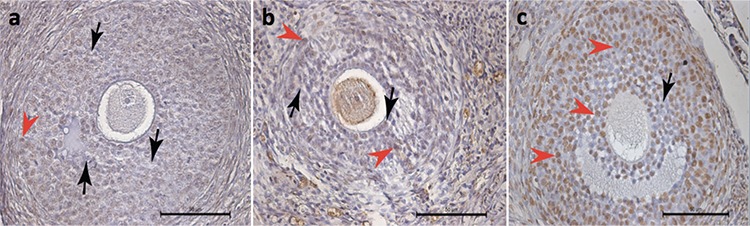
“>” denotes apoptotic granulosa cells of secondary follicles in histologic sections of the rat ovaries stained with ApopTag (brown stain), “>” denotes non-stained granulosa cells of the secondary follicles; a) control group, b) Pomeroy group, c) bipolar electrocauterization group (TUNEL assay x400)

**Figure 4:**
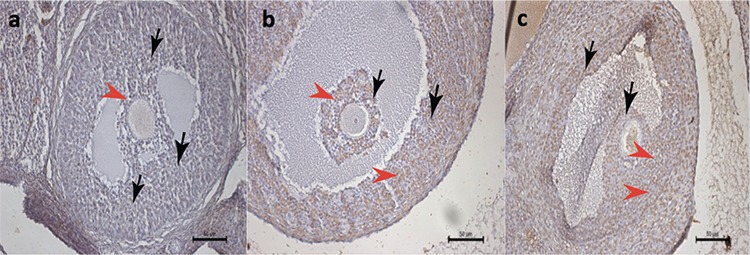
“>” denotes apoptotic granulosa cells of tertiary follicles in histologic sections of the rat ovaries stained with ApopTag (brown stain), “>” denotes non-stained granulosa cells of the tertiary follicles; a) control group, b) Pomeroy group, c) bipolar electrocauterization group (TUNEL assay x200)

On the other hand, the apoptotic indexes in secondary (Figure 3) and tertiary (Figure 4) follicles were similar in each study group (p=0.237 and p=0.069, respectively, Table 1).

## Discussion

Menstrual disorders such as prolonged or frequent bleeding, spotting, and dysmenorrhea have been observed after tubal sterilization procedures and these symptoms have been evaluated under the heading *“Post-tubal ligation syndrome”*. The effects of surgical female sterilization and whether the method of surgical sterilization per se has an effect on ovarian function have been investigated for years, yet there is still no consensus on this topic and study results are conflicting. We found that bipolar electrocauterization was associated with an increased apoptotic index of tubal epithelium and primary follicles, whereas Pomeroy’s technique had no effect on the apoptotic index of any follicle types or tubal epithelium.

There are two theories for the potential effects of surgery on ovarian function. First, tubal surgery may damage the vascular and neuronal structures within the mesosalpinx, which in turn may lead to cessation of paracrine/endocrine factors or nervous stimuli from the uterus to the ovaries (5). Second, the surgical procedure may cause impaired ovarian blood flow and diminished ovarian reserve (3). Based upon these theories, studies have been designed and several conflicting results have been reported (8).

Following tubal sterilization, a decrease in ovarian function and reserve has been demonstrated by measuring mid-luteal plasma progesterone, (4) follicle-stimulating hormone (FSH), luteinizing hormone (LH), and estradiol (5). In some other studies, it was reported that the decreasing ovarian function was associated with disturbed utero-ovarian blood flow/hypoxia subsequent to the surgical procedure (3,9,10,11). In contrast to these studies, it has also been reported that tubal sterilization and/or the method of sterilization per se had no effect on ovarian function. Garza-Flores et al. (12) reported no difference in the levels of mid-luteal estradiol and progesterone before and after tubal sterilization. Wu et al. (13) compared FSH, LH, prolactin, estradiol, and progesterone levels between women with tubal sterilization and controls and they found no difference between the two groups. There are also two Doppler studies reporting no alteration in utero-ovarian blood flow after tubal sterilization (8,14).

In addition to human studies, there are few animal studies regarding the effects of surgical sterilization on ovarian function and morphology. Riedel et al. (15) reported that the type of sterilization was related to ovarian function in rabbits, and animals sterilized with unipolar electrocauterization had significantly lower levels of progesterone compared with the bipolar electrocauterization group and control group. Zhao et al. (16) performed a study on monkeys and reported no difference in progesterone levels after salpingectomy. In addition to hormonal assays, there are also studies assessing the effect of surgical sterilization on the histologic findings of the ovaries (11,14,17). Ovarian morphology of rabbits was evaluated and a significant reduction of tertiary follicle numbers per ovary in the postoperative 3rd month was reported, irrespective of the sterilization procedure (17). Kuscu et al. (18) investigated the late effects of sterilization on ovarian histology in rats. They concluded that the median number of healthy tertiary follicles per rat had been significantly reduced when compared with controls, regardless of the method used for sterilization. Nevertheless, we found no difference between the apoptotic index of the secondary and tertiary follicles regardless of the tubal sterilization method.

In the present study, we found that the apoptotic indexes in the tubal epithelium and primary follicles of ovaries were increased in the bipolar electrocauterization group compared with the control and Pomeroy’s technique groups. In human studies, it has been previously reported that tubal sterilization with the modified Pomeroy’s technique was neither associated with a decrease in ovarian reserve nor with an adverse effect on the blood supply of ovarian stroma when compared with healthy women without surgery (19,20) and that tubal sterilization through bipolar electrocoagulation was most likely to have an adverse effect on the ovarian reserve in the postoperative period compared with mechanical clips (21). However, to the best of our knowledge, this is the first study to compare the apoptotic index in the fallopian tubes and ovaries vis-à-vis two surgical contraception methods.

The apoptotic index detected in both surgical methods may have an impact on long-term functions of ovaries and tubes. It is an important issue when we take into account patient requests for tubal reversal. There are only a few studies have investigated the predictive factors of tubal reversal success (22,23), and it is yet not possible to draw exact conclusions from the available data (24). From this point of view, one might speculate that the higher apoptotic index of the tubal epithelium in bipolar electrocauterization might be related with a lower success rate of tubal reversal.

There is an ongoing debate regarding ovarian cancer prevention with tubal ligation. Although a recent meta-analysis showed a 34% reduction in the risk of epithelial ovarian cancer with tubal ligation (25), in the general population, it is recommended to perform a bilateral salpingectomy instead of tubal ligation for patients who would like to have a tubal sterilization with the belief of better cancer prevention and contraception (26). In this context, further studies are warranted to compare the effect of salpingectomy and tubal ligation only on the ovary. 

There are several limitations to the current study. The main limitation is the confounding effect of a natural ovarian cycle. Follicles that would not progress to ovulation would naturally undergo apoptosis, so this fact should be kept in mind while interpreting the results of the study. However, we designed the study with a control group in order to eliminate this possible confounder. The other limitations of the current study are the lack of monitoring of anti-Müllerian hormone levels to investigate the ovarian reserve and lack of a long-term follow-up period to compare the menopausal onset time of the rats. One should bear in mind that even the findings of current study were against using bipolar electrocauterization in tubal ligation; the study results should be confirmed by randomized, controlled, long-term human studies to determine whether there is clinical relevance to our significantly different findings. 

In conclusion, tubal sterilization with Pomeroy’s technique, as a permanent contraception method, revealed a lower apoptotic index when compared with tubal sterilization with bipolar electrocauterization.

## Figures and Tables

**Table 1:**
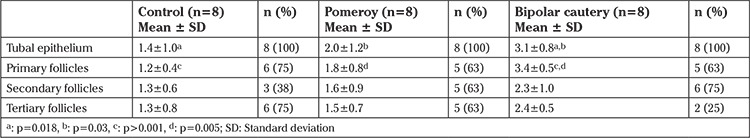
The numbers of follicle types in each group and the mean apoptotic index of each follicle type
